# Supplementary figures and images for: The application of artificial intelligence to support biliary atresia screening by ultrasound images: A study based on deep learning models
Source: PLoS One. 2022 Oct 19;17(10):e0276278. doi: 10.1371/journal.pone.0276278 (PMC9581370; doi:10.1371/journal.pone.0276278)

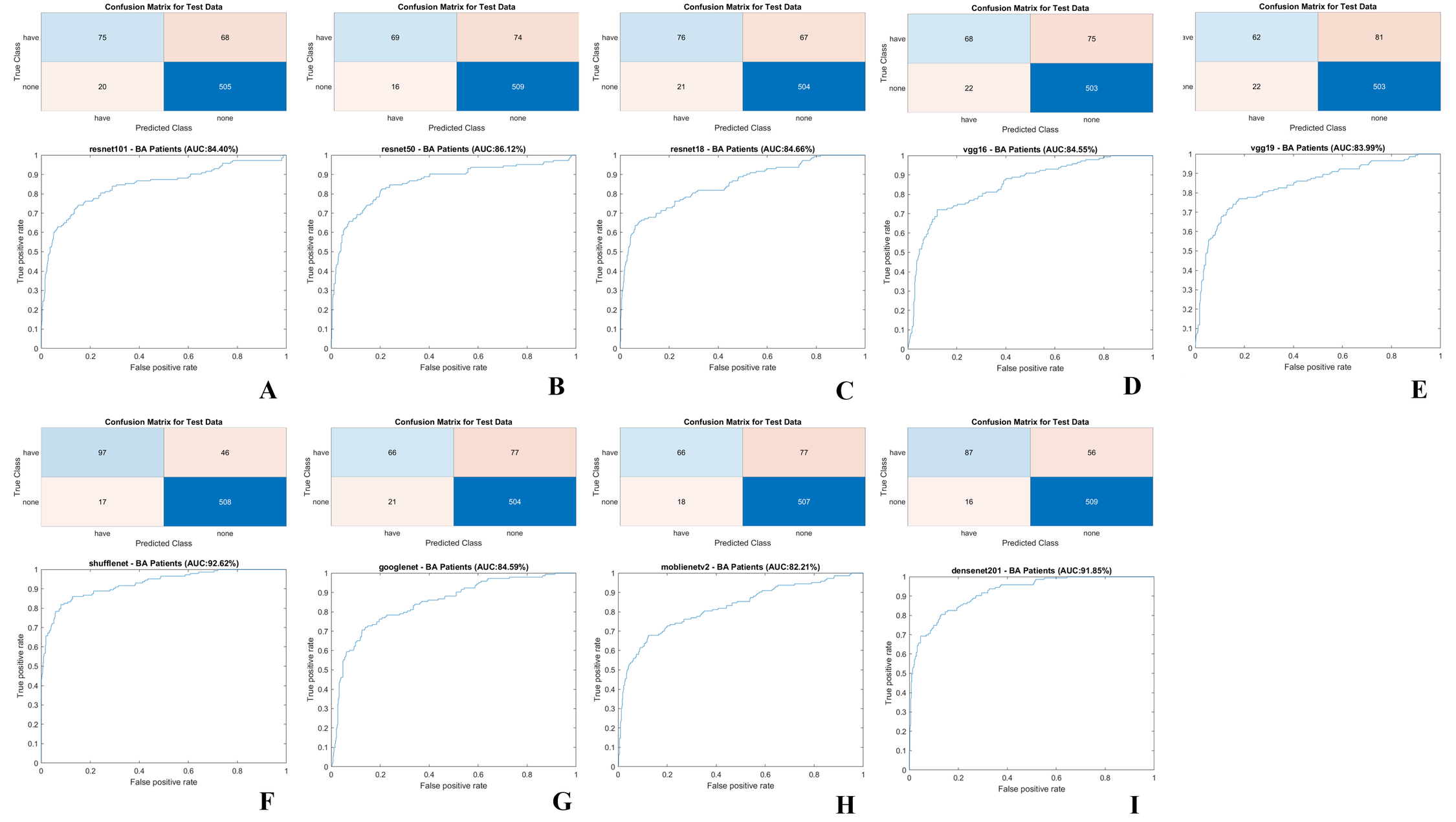

Supplement: S1 Fig — A, Confusion matrix and ROC curve of ResNet-101. B, Confusion matrix and ROC curve ResNet-50. C, Confusion matrix and ROC curve ResNet-18. D, Confusion matrix and ROC curve of VGG-16. E, Confusion matrix and ROC curve of VGG-19. F, Confusion matrix and ROC curve of ShuffleNet. G, Confusion matrix and ROC curve of GoogleNet. H, Confusion matrix and ROC curve of MobileNetV2. I, Confusion matrix and ROC curve of DenseNet-201. AUC: area under curve. (TIF) [file pone.0276278.s001.tif]

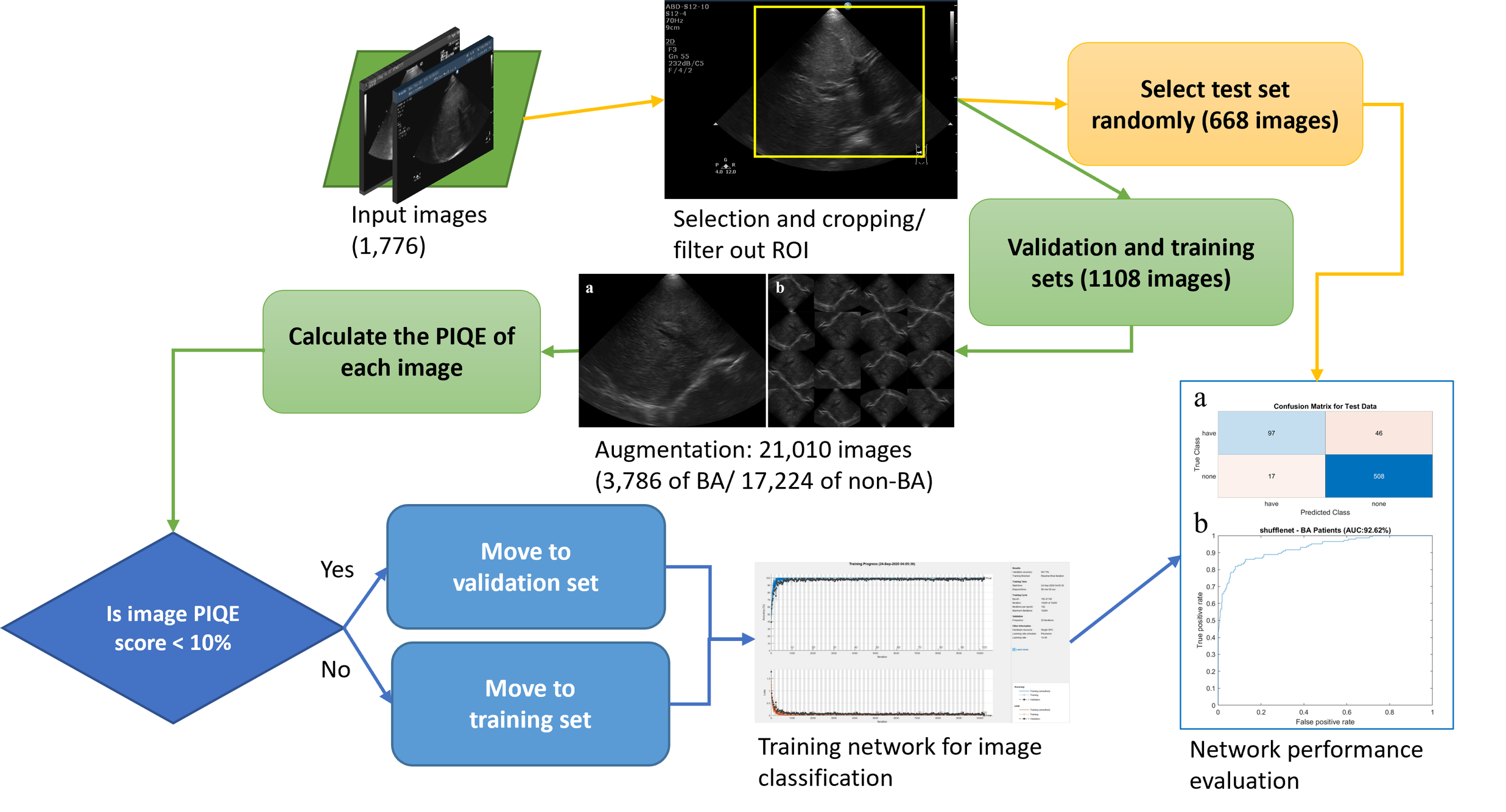

Supplement: S1 Graphical abstract — (TIF) [file pone.0276278.s004.tif]
