# Supplementary material for: The application of artificial intelligence to support biliary atresia screening by ultrasound images: A study based on deep learning models
Source: PLoS One. 2022 Oct 19;17(10):e0276278. doi: 10.1371/journal.pone.0276278 (PMC9581370; doi:10.1371/journal.pone.0276278)
Supplement: S1 Table — The p values in bold texts indicate significant difference. (DOCX) [file pone.0276278.s002.docx]

**S1 Table Comparison of AUC between models by Delong Test.**

| Models  (AUC) | ResNet-101  (0.8440) | ResNet-50  (0.8611) | ResNet-18  (0.8466) | VGG-16  (0.8455) | VGG-19  (0.8399) | ShuffleNet  (0.9262) | GoogleNet  (0.8459) | MobileNetV2  (0.8221) | DenseNet-201  (0.9185) |
| --- | --- | --- | --- | --- | --- | --- | --- | --- | --- |
| ResNet-101  (0.8440) |  | 0.5623 | 0.9311 | 0.9602 | 0.8925 | **0.0018** | 0.9496 | 0.4797 | **0.0054** |
| ResNet-50  (0.8611) |  |  | 0.6220 | 0.5964 | 0.4750 | **0.0109** | 0.6057 | 0.1984 | **0.0274** |
| ResNet-18  (0.8466) |  |  |  | 0.9708 | 0.8247 | **0.0024** | 0.9814 | 0.4277 | **0.0070** |
| VGG-16  (0.8455) |  |  |  |  | 0.8533 | **0.0021** | 0.9893 | 0.4493 | **0.0063** |
| VGG-19  (0.8399) |  |  |  |  |  | **0.0011** | 0.8429 | 0.5675 | **0.0035** |
| ShuffleNet  (0.9262) |  |  |  |  |  |  | **0.0022** | **0.0001** | 0.7308 |
| GoogleNet  (0.8459) |  |  |  |  |  |  |  | 0.4414 | **0.0065** |
| MobileNetV2  (0.8221) |  |  |  |  |  |  |  |  | **0.0004** |
| DenseNet-201  (0.9185) |  |  |  |  |  |  |  |  |  |

The *p* values in bold texts indicate significant difference.
