# Supplementary material for: The application of artificial intelligence to support biliary atresia screening by ultrasound images: A study based on deep learning models
Source: PLoS One. 2022 Oct 19;17(10):e0276278. doi: 10.1371/journal.pone.0276278 (PMC9581370; doi:10.1371/journal.pone.0276278)
Supplement: S1 File — Details of execution results are described. (DOCX) [file pone.0276278.s003.docx]

**Execution results of ResNet**

In the field of image classification, the residual network (ResNet) is often the first choice. ResNet was proposed in 2015, first published in 2016. Because of its simple and practically deeper architecture, many works of literature in different fields of classification, detection, or segmentation were based on ResNet. Compared with AlexNet, VGGNet, and GoogLeNet, ResNet has proven that as the depth of the network increases, the accuracy and other indicators of the network should be increased instead of deteriorating or gradient vanishing. For the backpropagation relationship during training the network, the gradient would be smaller than the previously hidden layers after expanding the depth of the network model. It means that the weight update of the previous layers would stagnate, causing the problem of gradient vanishing. In addition, the deeper network usually means a more significant number of parameters and more complex learning and optimization, resulting in a gradual decrease in training accuracy. ResNet adopts a design structure like the recurrent neural network (RNN) to overcome information loss. Take ResNet-50 as an example; after every three layers (1×1, 3×3, 1×1), the output value and the direct contact channel of the input value establish a bottleneck-like convolution method to ensure that the shallow information can be retained after processing by the multi-layer neural network. Therefore, the number of parameters needed to be calculated is reduced.

US image-based test sets for the overall evaluation and BA diagnosis of the ResNet network models were illustrated (S1 Fig A-C). The experimental results revealed that using the same training set, validation set, and training parameters, the evaluation indicators of the three different depths of the ResNet network were not significantly different. Generally, as the network gets deeper, the model’s accuracy should increase accordingly. However, according to the AUC of ROC curve, ResNet-50 showed the best result considering individual US images in this study.

**Execution results of VGGNet**

The confusion matrix and ROC curve of the VGGNet network models for BA diagnosis were illustrated (S1 Fig D, E). The network size of VGG-16 and VGG-19 was several times larger than other networks. However, the execution results of the test sets were not significantly better than other networks. The AUC of VGG-16 and VGG-19 were 0.845 and 0.839, respectively. The models might be able to exert better results on integrated learning.

**Execution results of ShuffleNet**

SqueezeNet is a lightweight network model proposed in 2016. The Fire module compression strategy makes the size of parameters only 5 MB, only 2.14% of AlexNet with the equivalent performance. The Squeeze-and-Excitation Networks (SENet) was subsequently proposed and won the ILSVRC 2017. The global average pooling (GAP) of SENet aggregates each feature map, strengthens the influence of essential feature maps, and weakens unnecessary feature maps. The strategy effectively improves the accuracy of output. In addition, the “Squeeze-and-Excitation” (SE) module structure is straightforward and can be easily deployed in most network models. ShuffleNet is based on SqueezeNet with some changes. The ShuffleNetV1 architecture refers to the ResNet bottleneck design. The group convolution and channel shuffle are used to compress calculations, exchange information between channels, and learn more complex features. Compared with ShuffleNetV1, ShuffleNetV2 uses “Concat” to replace “Add” in V1, avoiding the increasing multiply-accumulate (MAC) of many 1×1 pointwise convolutions.

The performance of the ShuffleNet model for US image-based BA diagnosis was shown. In repeated experiments, ShuffleNet had shown good classification performance in diagnosing BA (S1 Fig F). AUC was as high as 0.926. Besides, the runtime was shorter than ResNet-101, ResNet-50, VGG-16, VGG-19, and DenseNet because of the lightweight. Though ShuffleNet was slower than GoogleNet and MobileNet in this study, the difference was only 0.59 s and 0.64 s, respectively.

**Execution results of GoogleNet**

GoogleNet (also known as InceptionNet) did not have superior test results (S1 Fig G). Within lightweight models, GoogleNet had the lowest accuracy, precision, sensitivity, and specificity. Overall, GoogleNet only had better performance than VGG-19. The results confirmed that simply increasing the breadth of the network model did not benefit the identification of BA patients based on US images.

**Execution results of MobileNet**

The concept of MobileNetV1 is to split convolution into two parts: depthwise convolution and pointwise convolution (1×1 convolution). To put it simply, it is to group the channels first; then, a 3×3 kernel is used to collect the spatial features of each channel. Then 1×1 convolution is used to generate additional features from these depthwise features from the depth direction. Such calculations would be less than half of the original convolution structure. The calculation amount of depthwise separable convolution is the sum of the calculation amount of both depthwise and pointwise convolutions, compared with the calculation amount of general convolution, which is the product of the two values. Therefore, the depthwise separable convolution can save more computation when the kernel map is more extensive. Depthwise separable convolution could be considered a particular form of grouped convolution. Grouped convolution was initially developed in AlexNet and was originally designed for limited hardware resources.

MobileNetV2 is based on MobileNetV1, and linear bottlenecks and inverted residuals are added. Unlike the traditional residual method of dimensionality reduction and dimensionality increase, the inverted residual method works oppositely. The linear activation functions (such as a sigmoid function) are used instead of rectified linear unit (ReLU) activation functions in the low-dimensional hidden layers to reduce the information loss caused by ReLU. As the benchmark for lightweight networks, the parameters of MobileNet were much more than ShuffleNet. The evaluation results were like VGGNet and GoogleNet (S1 Fig H). The AUC of MobileNetV2 was the lowest among network models, only 0.822. The only advantage of the network was the shortest execution time.

**Execution results of DenseNet**

The results of DenseNet-201 were shown (S1 Fig I). Theoretically, DenseNet had advantages over ResNet by enhancing the propagation of features, obtaining more original features of each layer, and reducing network parameters. DenseNet-201 revealed the lowest FPR and the second-best AUC in the evaluation results. However, the training time, execution time, and actual parameter amount in this study were the weakness of this network model.

**Comparison between models by Delong Tests based on AUC**

Delong test was used to compare the performance of each model and the results were listed in S1 Table. ShuffleNet achieved the best performance by AUC and DenseNet was the second best. The two models outperformed all others by significant differences. There was no difference between the two models (*p* = 0.7308). However, DenseNet requested nearly four times of runtime as ShuffleNet.
